# Diabetes Diamantina Community: a tool to promote communication and education in diabetes

**DOI:** 10.1186/1758-5996-7-S1-A175

**Published:** 2015-11-11

**Authors:** Edson da Silva, Juliana Sales Rodrigues Costa, Eduardo Augusto Barbosa Figueiredo, Mayara Dumont Cunha, Daniela Pereira de Castro, Marileila Marques Toledo, Maryane Oliveira Campos, Luciana de Freitas Campos

**Affiliations:** 1 Universidade Federal dos Vales do Jequitinhonha E Mucuri-UFVJM, Diamantina, Brazil

## Background

Social networking sites (SNS) like Facebook represent a common place to seek information, but very little is known about the representation and use of health content on SNS. In recent yrs., people with diabetes have taken by the millions to diabetes SNS and blogs as well as main stream social media platforms, like Twitter and Facebook, to connect with their peers in ways that were impossible before. However, these sites vary considerably in quality and authenticity of the content, which is dangerous for diabetics.

## Objectives

The aims of the study were to evaluate the characteristics of the Diabetes Diamantina, an active Facebook diabetes community, as strategies to improve communication and education in diabetes.

## Materials and methods

Diabetes Diamantina Community (DDC) was founded on May 16, 2015. In this exploratory study, we took a sample of all messages posted daily on the DDC during 30 days in May and June 2015 to identify the general characteristics of this community. Messages were created by DDC administrator or shared the Fan Pages of major institutions of diabetes as SBD, ADA, IDF, ADJ and others. Posts were abstracted and aggregated using the Facebook database. Two investigators analyzed the posts and applied the Facebook codes to the data.

## Results

The results revealed that the community was international in nature. Its members were from Brazil (50%) and other 44 countries (50%). In the study period, 98 messages were posted on the DDC in Portuguese (n=49), English (n=29) and Spanish (n=20) languages. The number of participants was increasing steadily with 664 “Likes” and a “total reach” of 10,141 users of Facebook. Among participants, the large majority (78%) were gender female, and (55%) with age group between 18-34 yrs. old. The community was shaped as a social network where peer users share health content, social support, report personal experience, cultivate companionship, and exert social influence.

## Conclusion

Over time, new technologies are likely to emerge and, with them, new and creative ways for patients to connect will become available. DDC can improve diabetes education and can provide a forum for reporting personal experiences, asking questions, and receiving direct feedback for people living with diabetes in different countries. Based on the positive results, we discussed future directions for research of the DDC in a highly connected world.

